# Rethinking the Relationships Between Time Perspectives and Well-Being: Four Hypothetical Models Conceptualizing the Dynamic Interplay Between Temporal Framing and Mechanisms Boosting Mental Well-Being

**DOI:** 10.3389/fpsyg.2020.01033

**Published:** 2020-06-05

**Authors:** Bozena Burzynska, Maciej Stolarski

**Affiliations:** ^1^Faculty of Psychology, SWPS University of Social Sciences and Humanities, Warsaw, Poland; ^2^Faculty of Psychology, University of Warsaw, Warsaw, Poland

**Keywords:** time perspective, well-being, gratitude, savoring the moment, prioritizing positivity

## Abstract

Time perspective (TP) is a central aspect of human daily psychological functioning, with a pronounced impact on human thoughts, feelings, and behaviors. The particular TP dimensions are strongly associated with a range of various mental well-being indicators and were shown to predict as much as 40% of their variance. However, the relationship between TPs and specific mechanisms that enhance mental well-being still requires further exploration. In the present article, we conceptually analyze a potential interplay of TPs and three well-confirmed well-being “boosters” (WBBs)—gratitude, savoring the moment, and prioritizing positivity—which may prove responsible for the vital effects of TP on mental well-being. Each of the “boosters” has a clear temporal anchoring: gratitude stems from the appreciation of the past, savoring the moment refers mainly to the experience of the present, and prioritizing positivity engages planning behaviors that require future focus. We propose four theoretical models to be verified in further experimental research. The first model, the trait-behavior model, proposes that trait TPs increase the tendency to use particular WBBs in order to increase mental well-being. The second model, referred to as the accumulation model, offers that TPs mediate between WBBs and mental well-being; and finally, a regular practice of a specific WBB develops a specific TP (e.g., exercising a gratitude intervention enhances past-positive TP). The third model, the feedback loop, suggests that WBBs and TPs strengthen one another and contribute to higher mental well-being. The last model, which can be called the match–mismatch model, presents the influence of WBBs on mental well-being, where a particular TP plays a role of a moderator (e.g., present-hedonistic TP moderates the relationship between savoring and well-being). Implications of potential confirmation of each of the models for theory and practice are also discussed.

“The happy life is this – to rejoice to thee, in thee, and for thee.”

St Augustine (1949)

## Introduction

Research on the role of time perspective (TP) in shaping various aspects of well-being has provided a vital body of evidence for pronounced associations among these domains (see, e.g., Drake et al., 2008; Boniwell et al., 2010; Cunningham et al., 2015). When analyzed at both the level of particular temporal dimensions (see Stolarski and Matthews, 2016) and the global index of temporal balance (Zhang et al., 2013), TP may explain as much as 40% of variance in various aspects of subjective well-being. The effects also remain robust when the effects of personality traits, such as extraversion or neuroticism, are controlled (Stolarski, 2016; Stolarski and Matthews, 2016). However, little is known about actual mechanisms that are responsible for the existence and robustness of these associations. Cunningham et al. (2015) introduced a dual-pathway model of associations between TPs and mental well-being, claiming that mechanisms underpinning the relationships can be divided into the following: (1) top-down regulation, that is, direct effects of temporal foci on experienced emotions and related positive and negative affective states and moods; and (2) bottom-up mechanisms, where TPs foster particular actions and behaviors (e.g., career success or healthy behaviors) that lead to various outcomes that remain vital for features of well-being (e.g., wealth and health). In the present paper, we attempt to conceptually analyze a specific group of behaviors that—owing to their temporal anchoring—appear to be conceptually justified candidate variables to explain the well-confirmed albeit poorly understood associations.

## Time Perspectives

As reported by Zimbardo and Boyd (2008), TP is “the often unconscious personal attitude that each of us holds toward time and the process whereby the continual flow of existence is bundled into time categories that help to give order, coherence, and meaning to our lives” (p. 51). Stolarski et al. (2018) claim that TPs should be considered both as a state and as a trait. State TP reflects temporary focus on one of three time horizons: the past, the present, or the future, occurring while continuously framing the present experience. On the other hand, trait TPs reflect stable, habitual tendencies to remain focused on a particular temporal perspective. These tendencies could be measured as trait-like dimensions. Following this conceptualization, Zimbardo and Boyd (1999) established a five-factor model of TPs with dimensions: past-negative, past-positive, present-hedonistic, present-fatalistic, and future. Past-negative implies focusing on and reminiscing about aversive or painful situations from one’s life. Past-positive reflects concentration on positive, good past events and pleasant memories. Present-hedonistic depicts seeking immediate pleasure, giving into the temptation, and indulging the senses. Present-fatalistic is marked by a negative and helpless attitude toward the future and current existence. Later theoretical considerations and empirical analyses of TP resulted in distinguishing between two prospectively oriented dimensions: future-positive and future-negative (Carelli et al., 2011). Future-positive depicts focus on personal goals and consideration of distant consequences of current behaviors. Future-negative implies worrying about the future and anticipating negative outcomes.

Empirical evidence suggests that TPs robustly contribute to the prediction of mental well-being (Romero et al., 2014). For example, Kazakina (1999) reported a positive relationship between positive affect and present orientation, as well as between past-positive TP and life satisfaction. Other research also provided evidence of a positive correlation between present-hedonistic and future dimensions and subjective well-being (Zaleski et al., 2001), optimism (Lennings, 2000), and life satisfaction (Drake et al., 2008). Zhang and Howell (2011) demonstrated that TP dimensions accounted for over one-third of variance in life satisfaction, with an incremental 14% over and above the Big Five personality traits. Additionally, negative TPs were shown to have a significant impact on mental well-being. Individuals with past-negative or present-fatalistic perspectives, for example, tend to be less satisfied with their lives and to experience less positive affect and more negative affect (Boniwell et al., 2010; Carelli et al., 2011). Interestingly, the effects were often markedly stronger than those obtained for the “positive” TP features. The results were replicated in multiple studies (e.g., Stolarski and Matthews, 2016), providing powerful support for the claims about the vital role of temporal framing in mental well-being (see Stolarski et al., 2018).

## Well-Being Boosters: Mechanisms That Improve Human Well-Being

Research conducted within the positive psychology framework (Seligman and Csikszentmihalyi, 2000; Tkach and Lyubomirsky, 2006) provided evidence for multiple factors that influence mental well-being. Researchers examined mental and behavioral features of happy people and identified several actions that may foster one’s mental well-being if systematically repeated (Lyubomirsky and Layous, 2013). In this article, we focus on three factors that were shown to vitally increase mental well-being: gratitude (Watkins et al., 2003; Wood et al., 2009); savoring (Quoidbach et al., 2010); and prioritizing positivity (Datu and King, 2016). They were selected based on two major criteria: (1) their impact on mental well-being had to be well-confirmed (see the discussion below); and (2) each of them had to have a clearly marked temporal anchoring that allowed us to link it conceptually with a focus on a particular time horizon.

### Gratitude

Gratitude is a complex construct, which can be defined as an emotion, an attitude, a moral virtue, a habit, and a personality trait, as well as a coping response (Emmons and McCullough, 2003). A clinical definition states that gratitude is “the appreciation of what is valuable and meaningful to oneself and represents a general state of thankfulness and/or appreciation” (Sansone and Sansone, 2010, p. 18). The definition enables us to understand gratitude not only as a reaction to receiving something from someone but also as being thankful for positive experiences such as a success at work or appreciation of various valuable life experiences.

According to the broaden-and-build theory, gratitude belongs to positive emotions that broaden a person’s momentary thought-action repertoire and build personal mental resources (Fredrickson, 2004). Research shows that positive emotions such as gratitude broaden cognition and behavioral abilities (Garland et al., 2010). With time, behavioral flexibility is developed, which in turn builds individual resources, such as mindfulness, resilience, and physical health (Waugh and Fredrickson, 2006; Cohn et al., 2009).

Gratitude is often mentioned as one of the crucial factors improving mental well-being (Emmons and Crumpler, 2000; Sansone and Sansone, 2010). It fosters positive feelings, which later add to one’s overall sense of mental well-being. Moreover, gratitude helps people to deal with difficult and stressful situations on a daily basis (Fredrickson, 2004) and improves physical and psychological health (Emmons and Stern, 2013). Gratitude also supports building high-quality relationships between a grateful person and the receiver of gratitude (Algoe et al., 2008) and fosters empathy and increases self-esteem (Chen and Wu, 2014). Nevertheless, the mechanism by which gratitude is associated with mental well-being is still unknown (Emmons and Mishra, 2011), and it seems significant to further explore that relationship.

### Savoring

Savoring is defined as a disposition to concentrate on and cherish past, present, and upcoming life events (Bryant, 2003). People differ in their capacity to savor positive outcomes, and thus savoring can be measured as an individual difference (Bryant, 1989). In research about savoring, the focus is put on beliefs about savoring as a separate form of anticipated control over positive emotions.

Bryant (2003) identified three components of savoring: savoring through anticipation; savoring through reminiscing; and savoring the moment. The components are time-oriented. The first, savoring through anticipation, concerns future orientation, where an individual experiences and relishes the positive emotions aroused by thinking about the future. Savoring through reminiscing relates to the positive emotions that occur when a person recalls past events and appreciates them. The last component, savoring the moment, relates to a situation when one experiences and cherishes positive emotions in response to the present. Savoring in a general sense, as an accumulation of the three components, has been positively linked to antecedents of subjective well-being such as gratification and self-esteem (Bryant, 2003), as well as greater work–life balance (Smith and Bryant, 2017).

Savoring the moment was chosen for the present consideration for several reasons. First, its definition partly overlaps with present-hedonistic TP. Both involve currently occurring experiences, and in both definitions, it is underlined that intensifying positive experiences remain their core feature. Savoring the moment can strengthen the effect of positive events (Bryant and Veroff, 2007). A person with a high level of present-hedonistic orientation focuses on the present moment, appreciates hedonistic pleasures, and seeks excitements and new, intense sensations; and thus, savoring appears to be an effective strategy for satisfying their needs. Second, present-hedonistic time orientation is correlated with high novelty and sensation seeking in the present (Boniwell and Zimbardo, 2004). Savoring the moment might provide methods that enhance enjoyment of pleasant experiences as they appear (Bryant and Veroff, 2007).

### Prioritizing Positivity

Prioritizing positivity is the tendency to structure daily life to include pleasant experiences (Catalino et al., 2014). It considers organizing individuals’ daily lives in a way that would maximize their experience of positive emotions and, in consequence, could lead to greater well-being. Prioritizing pleasant experiences was found to be effective in pursuing positive emotions (Catalino et al., 2014). The method is an application of Gross’ (2015) extended process model of emotion regulation, which describes five diverse families of regulatory processes. One of them is situation selection. This refers to taking steps to select a situation to which an individual will be exposed. In the case of prioritizing positivity, it would refer to planning one’s own actions, aiming to maximize probability of positive, pleasurable life events (Gross and Thompson, 2007). As a consequence, one can purposely seek out circumstances that possibly give rise to positive emotions. Prioritizing positivity is related to various beneficial well-being indicators, which range from more regular positive emotions to lesser depressive symptoms (Catalino et al., 2014; Van Cappellen et al., 2018).

Mauss et al. (2011) showed that the explicit pursuit of happiness, regarded as the key component of mental well-being, may backfire when a person focuses on maximizing positivity in the moment. Some research suggests a paradoxical effect: the more people pursue positive emotions, the less likely they are to experience positive outcomes that improve their level of happiness (Mauss et al., 2011). Also, the capacity to withdraw from unreachable goals is a crucial aspect of psychological health, and it is recommended to remove high standards for happiness if they are disengaging (Wrosch et al., 2003). On the other hand, evidence suggests that individuals who seek happiness by looking for circumstances in which they can possibly experience positive emotions could thus recap accidental and life-sustaining awards (Van Cappellen et al., 2018). It might be profitable to identify and purposely engage in activities that increase mental well-being (Ford and Mauss, 2014). Prioritizing positivity seems to follow the “rules” advised to avoid the potential backfiring from impulsively pursuing positive emotions.

## Positive Psychology of Time Perspectives

Taking into account both the major assumptions of TP theory and the robust body of research carried out to date, temporal framing of personal experiences appears to be a vital self-regulatory mechanism fostering various aspects personal adaptation and consequently influencing mental well-being (Stolarski et al., 2015a, 2018). The way in which individuals deal with their own temporality may exert powerful influences both on emotional responses to a given situation and, partially as a consequence of accumulation of such responses, on global assessments of life satisfaction (see Stolarski and Matthews, 2016).

According to the dual-pathway model introduced by Cunningham et al. (2015), the effects may be either direct (e.g., via direct regulation of emotional states and influences on appraisals of previous, ongoing, and anticipated life events) or mediated via other antecedents of mental well-being, resulting from TPs (such as health or education). For instance, the habitual tendency to interpret difficult life events as possibilities to develop novel competences and develop oneself (characteristic for past-positive TP) has completely different consequences for one’s mood and mental well-being than viewing such situations as sources of pure trauma (typical for past-negative). This is an example of a top-down, cognitive-regulatory role of TP in emotional functioning, where the influences of temporal perspectives take place more or less directly, being mediated mainly by cognitive and emotional appraisals of perceived reality (see Cunningham et al., 2015). At the same time, individual differences in TPs naturally influence our actions across a variety of life domains, such as health behaviors (Daugherty and Brase, 2010); risk taking (Jochemczyk et al., 2017); romantic relationships (Stolarski et al., 2016b); or aggression (Stolarski et al., 2016c). Consequences of such actions (e.g., good health and fitness vs. illnesses and obesity, career success vs. failure, building supportive social networks vs. social exclusion, or even imprisonment) are crucial for experiencing happiness and global assessments of life satisfaction. In this bottom-up path of TPs’ influence on mental well-being (see Cunningham et al., 2015), effects of temporal framing are mediated via everyday life situations, and thus this pathway may be treated as an indirect one. Interestingly, particular dimensions of TP identified by Zimbardo and Boyd (1999) may be more important for the former pathway, whereas other ones seem central for the latter: In light of multiple studies carried out to date, past dimensions exert pronounced influences on experienced emotions and appraisal of ongoing events, as well as expectations formulated toward the future (Stolarski et al., 2014), while actual behaviors are much more important for actual behaviors that may influence mental well-being indirectly (see, e.g., Keough et al., 1999; Daugherty and Brase, 2010).

A review of the nature of the three well-being boosters (WBBs) described above (i.e., gratitude, savoring the moment, and prioritizing positivity) highlights a specific temporal anchoring of each of them. For instance, Lewin (1942) emphasized that “setting up of goals is closely related to (future) TP” (p. 80). At the same time, goal setting is essential for prioritizing positivity (Catalino et al., 2014). Therefore, it seems justified to expect a direct link between the tendency to focus on future consequences of one’s own behavior and giving priority to those behaviors that should foster one’s mental well-being in the future. Moreover, given that high intention – behavior consistency is characteristic for future-oriented individuals (Van Ittersum, 2012), the effect of prioritizing positivity on mental well-being could be enhanced by this temporal perspective. Future-negative TP, in contrast, would be negatively related to planning pleasant activities. It refers to a pessimistic attitude toward upcoming events and a concentration on anticipated failure to plan efficiently (Carelli et al., 2011). Therefore, focusing on the negative aspects of the future would plausibly act as an inhibitor of setting satisfying goals and becoming involved in enjoyable activities.

Analogical connotation may be expected with respect to present focus and savoring. The ability to remain focused on the present experience appears to be an essential component of savoring. On the other hand, pleasurable benefits from savoring seem of particular importance for individuals manifesting elevated levels of the present-hedonistic perspective, particularly taking into account their habitual striving for maximizing pleasure and pronounced sensation seeking (Zimbardo and Boyd, 1999). Therefore, savoring the moment seems to be a WBB characteristic for individuals with present-hedonistic TP. At the same time, individuals with an elevated present-fatalistic orientation might experience difficulties with savoring. They manifest a hopeless attitude toward life and might not be capable of savoring the positive events that they experience. Such differences in the capacity to savor positive experiences might subsequently impact mental well-being (Bryant, 2003).

One may argue that the concept of mindfulness could be included in our model instead of savoring, particularly taking into account its well-established associations with both TP and mental well-being (Drake et al., 2008; Stolarski et al., 2016a). Both mindfulness and savoring the moment concern the current experience. Nevertheless, the constructs are qualitatively different, and when it comes to shaping daily positive emotions, they play a complementary role (Kiken et al., 2017). Mindfulness is characterized as an inherent state of consciousness that includes consciously attending to one’s present experience (Brown and Ryan, 2003). The attention can be placed on any thought, emotion, or experience, be it positive, negative, or neutral (Hurley and Kwon, 2012). Savoring the moment, in contrast, focuses on positive emotions, and it aims to strengthen or prolong positive emotions, whereas mindfulness relates to general experiences, without necessarily increasing positive emotions (Hurley and Kwon, 2012). Given that the present-hedonistic orientation depicts striving for positive, pleasurable experiences and that it remains associated with higher impulsivity (Zimbardo and Boyd, 1999), it seems justified to expect that savoring, rather than mindfulness, remains the actual link between the present-hedonistic orientation and mental well-being, particularly taking into account the negative associations between mindfulness and impulsivity (Peters et al., 2011).

Finally, past-positive seems to share some central features with gratitude. People tend to be grateful for things that have already happened, and frequently recalling pleasant memories naturally helps them to realize more reasons to be grateful. A recent paper by Zhang (2020) provides evidence for the fact that grateful individuals are happier because they tend to develop greater levels of past-positive TP. Again, an opposing causality also seems plausible: frequent experiences of being grateful may become accumulated and “crystallized” in a generalized positive view of the personal past. Past-negative TP, in contrast, manifests in a limited capacity to concentrate on positive aspects of life and highlights what causes discomfort and pain (Stolarski et al., 2018). The resulting bitterness and negative evaluation of life might lead to a markedly diminished experience of gratitude.

**FIGURE 1:**
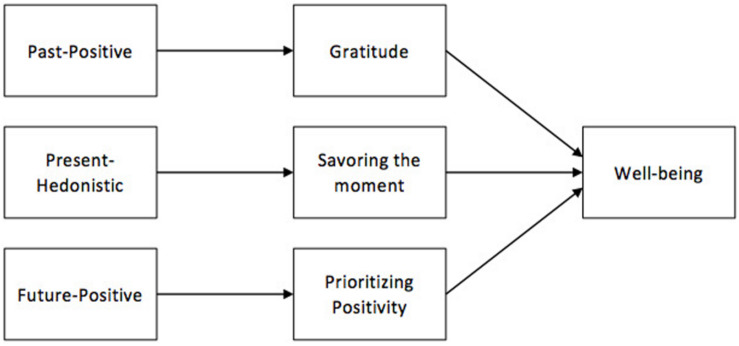
The general conceptual model illustrating the hypothesized effects of time perspectives on well-being boosting behaviors (WBBs; which are gratitude, savoring the moment, and prioritizing positivity) and mental well-being.

Each of the three potential interplays between particular TPs and WBBs may uniquely contribute to the experience of mental well-being. Therefore, our general conceptual model presented in Figure 1 comprises the three major hypothetical TP – WBB dyads that may at least partially uncover the essence of the incremental effects of particular TPs on mental well-being (e.g., Zhang and Howell, 2011). It is important to note that the direction of associations on the presented scheme remains to be determined (see the next paragraph discussing diverse hypothetical interplays between TP and WBBs), whereas this general illustration only focuses on identifying said dyads and underlining their role in shaping mental well-being.

To provide a broader conceptual anchoring for the present consideration, it seems vital to take into account major assumptions of construal level theory (CLT; Trope and Liberman, 2010). CLT posits that individuals directly experience only the here and now, and they are able to transcend that point by forming abstract mental construals of distal objects. Hence, even if people cannot experience what is not present, they can remember the past or make predictions about the future and speculate on what might have been. Research shows that diverse distances—temporal, social, spatial, and hypotheticality—are cognitively related to one another: thinking of a happening as distant on one dimension leads an individual to think about it as distant on other dimensions. Also, the diverse distances influence and are influenced by the level of mental construal currently “active.” For example, people use more abstract, simple, and coherent mental models (so-called high-level construals) to represent information about distant future events than information about near-future events (that are concrete, complex, and incoherent) (Trope and Liberman, 2003).

Self-distancing theory states that there are two main perspectives that individuals use when assessing a negative situation: self-distanced, which means “taking a step back” from the experience so a person can work through it more effectively (i.e., from the perspective of a fly on the wall); or self-immersed, which means visualizing events happening to a person all over again through one’s own eyes (Kross and Ayduk, 2016). Recent research shows that self-distancing plays an important role in facilitating adaptive self-reflection, mainly by enhancing a person’s level of psychological distance from the self, so that individuals tend to be increasingly capable of reasoning effectively about their own problems (Kross and Ayduk, 2016). Psychological distance helps to see the “big picture” rather than focus on details (Liberman and Trope, 2003). Importantly, Maglio et al. (2015) point out that temporal construal may in fact be another way to conceptualize state-like TP foci (Stolarski et al., 2018), with present TP reflecting more concrete construal and past and future TPs depicting abstract construals. Switching between now and then, between abstract and concrete, may thus reflect the process of a balanced time perspective (BTP) (Zimbardo and Boyd, 1999). Temporal construal could also provide a vital approach to elicit a shift in situational temporal focus and may be considered in future research aiming to test experimentally for the effects of situationally taken temporal perspectives on happiness and mental well-being.

## Four Conceptual Models Illustrating Potential Interplay Between Time Perspectives and Well-Being Boosters in Predicting Mental Well-Being

Multiple studies carried out to date provide evidence for pronounced effects of both particular TP dimensions and a balanced temporal profile of mental well-being. The WBBs discussed above seem to be natural candidate variables for the mechanisms linking TP with mental well-being, at least at the theoretical level. In the following part of this paper, we propose four models that illustrate conceptually possible patterns of dynamics between TPs and WBBs in predicting mental well-being. These models may be empirically verified in future research. Taking into account the potential complexity of mentioned relationships, it seems plausible that more than one of these models will find empirical support (i.e., different models will find empirical support for particular TP – WBB dyads, or even two mechanisms—e.g., mediation and interaction—may turn out to be supported within one dyad). Also, it is possible that some of the WBBs are in fact linked to more than one TP, depending on the context. For example, gratitude can relate not only to the past-positive perspective but also to the future-positive perspective when it comes to making intertemporal choices (DeSteno, 2009; DeSteno et al., 2014). The latter effect could stem from a projection of gratitude onto future expectations (inverse effects are possible for past-negative; see Stolarski et al., 2014). However, we predict that the TP – WBB associations will be the most pronounced within each of the said dyads.

### Model 1: Trait-Behavior Model

**FIGURE 2:**
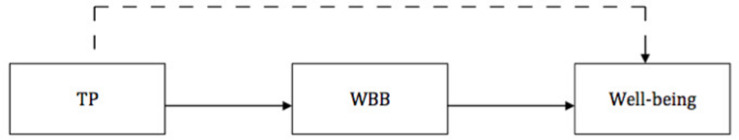
The trait-behavior model; TP, times perspective; WBB, well-being booster. Dashed line indicates that mechanisms other than given WBB may in parallel mediate the effect.

Model 1 stems from understanding TPs as relatively stable individual differences in habitual use of time horizons, including a particular attitude component such as positive or negative components (Stolarski et al., 2018), and seems to be the most intuitive among the hypothesized models. It assumes that trait TPs may impact the frequency of undertaking specific WBBs, indirectly leading to elevation or decrease of mental well-being (see Figure 2).

For instance, the future-positive perspective would plausibly increase the probability of taking into account possible future selves (Markus and Nurius, 1986) and could direct current activity to approach the desired selves and avoid the undesired selves. One of the central features comprising the vision of the possible self is the broadly understood well-being of the imagined future self. Such a vision should naturally lead to enhancement of the current and planned actions that lead to the realization of desirable self-related goals and maximization of positive experiences. The prioritizing positivity concept seems to fit this description perfectly: if people want to nurture their future mental well-being, they need to plan pleasant activities that evoke positive emotions (Quoidbach et al., 2009). The future-negative perspective, in contrast, could possibly inhibit the prioritization of positivity and enhance avoidance motivations, which, in consequence, would lead to lower well-being. The present-hedonistic focus, by definition, manifests in the motivation to strive for pleasure and excitement (Zimbardo and Boyd, 1999); thus, savoring the present moment seems to be the most natural way to satisfy these endeavors and should be more frequent among individuals with particularly high levels of this positive TP dimension. Focusing on the present experience is also characteristic of present-fatalistic TP, referring to a helpless and hopeless attitude toward life (Zimbardo and Boyd, 1999), and may play a greater role in maladaptive psychological outcomes such as tense arousal (Stolarski et al., 2014). We predict that this perspective may suppress savoring the moment and, as a result, might decrease mental well-being. Finally, the habitual tendency to recollect positive events from the past, essential for past-positive individuals, should lead to an elevated tendency to experience and practice gratitude (see Zhang, 2020), which remains one of the strongest prerequisites for the development of mental well-being (Wood et al., 2010). Remembering the past as distressful, which is characteristic of a past-negative perspective, might, in contrast, cause difficulties with feeling grateful. To sum up, within this model, TPs influence specific behaviors and actions that, in turn, impact one’s mental well-being.

### Model 2: Accumulation Model

The second model proposes that a regular practice of a given WBB influences respective TP and consequently leads to higher mental well-being. Zimbardo and Boyd (2008) have emphasized that despite the relative stability of habitual temporal foci, the individual TP profile remains under constant influences of ongoing life events, as well as personal, social, and cultural factors. Following this line of reasoning, we assume that repeatedly undertaken WBBs may enhance respective temporal focus. For instance, regularly exercising the prioritizing positivity intervention may lead to an increase in future-positive orientation, and consequently, it may contribute to higher mental well-being via enhancing optimistic positive expectations toward one’s personal future, which remains essential for this TP. Similarly, regular gratitude practice (e.g., writing gratitude letters; Boehm and Lyubomirsky, 2009) may lead to a reinforcement and accumulation of positive memories (see Zhang, 2020) and, consequently, an elevation of a past-positive temporal focus, ultimately leading to greater mental well-being. This model also suggests that regular practice of WBBs could change the focus from a negative to positive perspective. Frequently repeated savoring of the moment, for instance, might lead to shifting the temporal focus from a present-fatalistic to present-hedonistic perspective. Similarly, exercising gratitude could lead to reappraisal of one’s personal past and result in a shift from past-negative to past-positive.

Hodges and Clifton (2004) endorse strengths-based development, which means enhancing and developing one’s existing features, including TPs. An example of the positive development approach can be positive interventions (Lyubomirsky and Layous, 2013). Positive interventions focus on strengths and development of positive qualities in order to improve well-being (Senf and Liau, 2013; Kaczmarek, 2016). We suggest that regular exercising of WBBs can impact state TP and, if such a situation is consistently repeated, produce a stable, internalized impact on the adaptive TP dimensions, subsequently leading to an improvement in mental well-being. This line of reasoning is fully consistent with major assumptions of TP-based coaching (Boniwell et al., 2014) and TP therapy (Sword et al., 2014)—approaches that assume that certain psychological interventions eliciting a positive shift in the TP profile may subsequently boost psychological health and mental well-being.

**FIGURE 3:**
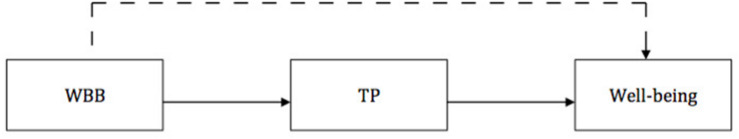
Accumulation model; WBB, well-being booster; TP, times perspective. Dashed line indicates that mechanisms other than given TP may in parallel mediate the effect.

This model is referred to as an accumulation model (see Figure 3). It depicts a situation where a specific behavior or group of behaviors (here: WBBs) result in an activation of a particular temporal perspective. If such an experience is consistently repeated, the respective temporal focus, through the process of habituation, becomes more available. The repeated WBB-induced temporal experiences become accumulated and exert a significant impact on mental well-being.

### Model 3: Feedback Loop Model

**FIGURE 4:**
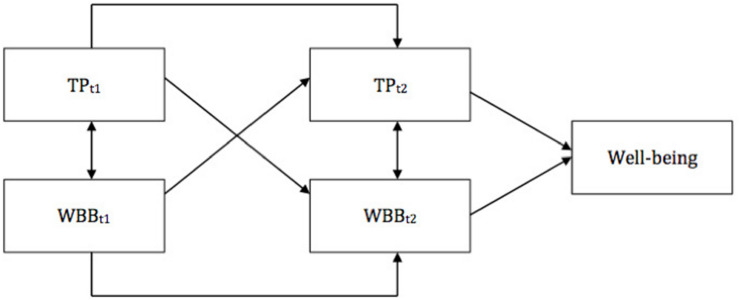
Feedback loop model. TP_*t1*_ and TP_*t2*_, time points for a particular time perspective; WBB_*t1*_ and WBB_*t2*_, time points for a particular well-being booster.

Can TPs lead to a sustainable increase in gratitude, savoring the moment, and prioritizing positivity? Alternatively, can particular WBBs have a significant impact on TPs? Two previous models bring some evidence that it might be unilaterally possible. The third model (see Figure 4) proposes a mutual relationship between the constructs: it suggests that WBBs and positive TPs strengthen one another and that such a reciprocal process contributes to higher mental well-being. The model can be described as a positive feedback loop: on the one hand, stable temporal tendencies manifested in one’s positive TP profile influence the probability of undertaking particular WBBs; on the other hand, frequent “visits” in a particular temporal horizon, resulting from undertaking a respective WBB, may produce a stable habit to make use of the TP. Evidence for the positive reciprocal influence of past-positive and gratitude, present-hedonistic and savoring the moment, and future-positive and prioritizing positivity would provide support for this model. Interestingly, if the feedback model is supported, it would also mean that the interplay of TPs and WBBs in shaping mental well-being is subject to the famous Matthew effect of accumulated advantage (Merton, 1968): a more adaptive temporal profile would lead to more frequent use of WBBs, and using WBBs would reciprocally foster adaptive temporal perspective, resulting in snowball-like elevation of mental well-being. Analogically, an inverse mechanism may be predicted for the negative TPs: maladaptive temporal foci may diminish WBB practices, which may lead to an accumulation of negative experiences and emotions. This, in turn, may result in undesirable shifts in the TP profile.

### Model 4: Match–Mismatch Model

**FIGURE 5:**
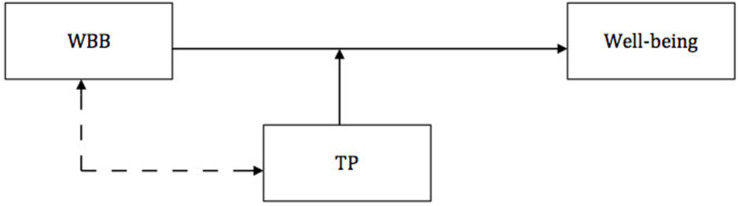
Match – mismatch model; TP, time perspective; WBB, well-being booster behavior. The dashed line indicates that a direct association between WBB and TP is possible but not necessary for the occurrence of an interaction effect.

The last model proposes that WBBs influence mental well-being, whereas TPs moderate these effects. Allemand et al. (2012) provided evidence for a moderating role of future TP on the relationship between forgiveness and mental well-being. In other studies, Sobol-Kwapinska (2016) and Stolarski (2016) showed that TPs may moderate well-established effects of personality on mental well-being. The research shows that the magnitude of associations between mental well-being and its well-established antecedents may depend on the level of particular TPs. The match–mismatch model (see Figure 5) extends this conclusion to the effects of WBBs on mental well-being: We believe that different levels of particular TPs may make respective WBBs more or less effective in their well-being-enhancing roles. Thus, the model proposes that if an elevated level of a particular TP corresponds with respective WBB, it shall facilitate its positive effects on mental well-being.

In the last few years, there has been a growing body of scientific evidence showing the contribution of gratitude to mental well-being (e.g., McCullough et al., 2001; Watkins et al., 2004). Emmons (2003) found that individuals are actually more successful at reaching goals when they consciously practice gratitude. The reason why gratitude interventions enhance mental well-being might be that gratitude enhances accessibility to positive memories (Emmons and Mishra, 2011). Grateful individuals are characterized by a positive memory bias (Watkins et al., 2004), and it has been shown that gratitude contributes to well-being by increasing the retrieval of positive memories (Emmons and Mishra, 2011). Given that individuals scoring high on past-positive orientation are characterized by more vivid, detailed, and accessible autobiographical memories (Ely and Mercurio, 2011), practicing and expressing gratitude may be easier and more natural and, therefore, exert a stronger positive impact on well-being. Accordingly, individuals with an elevated past-negative TP might experience greater difficulty experiencing and practicing gratitude, which may lead to diminished well-being.

Maximizing experienced pleasure is particularly important for individuals with elevated levels of present-hedonistic orientation (Zimbardo and Boyd, 1999, 2008). Savoring skills may provide such individuals with enhanced satisfaction of this sensation-seeking need. On the other hand, the positive affective consequences of savoring the present may be far less gratifying for individuals with low present-hedonistic orientation, as they do not value hedonically pleasant experiences as much as their counterparts. On the other hand, elevated levels of present-fatalistic TP may lead to the suppression of savoring positive events owing to a greater sense of helplessness and external locus of control.

Finally, the positive effects of prioritizing positivity on mental well-being (Catalino et al., 2014) may be elevated if an individual habitually uses the distant future horizon. First, this occurs because for people who are not accustomed to making plans and considering distant consequences of their behaviors, prioritizing positivity may turn out to be a difficult and unnatural behavior. Second, this also occurs because individuals with a broad future horizon and who are accustomed to performing mental time travel may be more capable of developing realistic and truly effective plans for including positivity-boosting actions in their daily plans. Finally, given their elevated intention – behavior consistency (Van Ittersum, 2012), they may be more effective in applying their well-being-boosting plans into their daily routine. An elevated future-negative TP, in contrast, may discourage individuals from setting up goals and planning pleasant activities. As a consequence, it might markedly decrease the effects of prioritizing positivity on mental well-being.

## Toward Empirical Verification of the Conceptual Models and Beyond: Future Research Pathways

The conceptual predictions introduced in this paper await empirical verification. Some of them (e.g., the claim regarding the interplay between past-positive and gratitude in predicting mental well-being) have recently gained initial support from empirical research (Zhang, 2020); however, most still remain speculative, and the question of which of the models proposed above reflect(s) actual dynamics between TPs, WBBs, and mental well-being remains unanswered.

Studies aiming to test the conceptual model certainly cannot be limited to the cross-sectional approach, which still remains the most commonly applied research design in current research on TP (see Stolarski et al., 2018). Although such an approach may be useful in providing an initial exploration of the presented ideas, it will not allow us to denominate causality in examined relationships. Thus, longitudinal and experimental approaches seem necessary to determine whether the general conceptual idea of temporal anchoring of WBBs is valid and, if it is, to answer the question of which of the alternative models depicting the interplay between WBBs and TPs in shaping mental well-being is the most accurate. The former approach, applying cross-lagged panel designs, would provide insight into the elusive issue of causality in TP – WBB dynamics. The latter would allow us to determine whether the magnitude of positive effects of the three positive WBB interventions on mental well-being indeed depends on individual levels of particular TPs. Another interesting idea concerns studies on the impact of interventions aiming to modify an individual’s temporal profile, such as TP coaching (Boniwell et al., 2014) or group training (Oyanadel et al., 2014), and on individuals’ tendencies to undertake WBB behaviors. If WBBs were to mediate between participation in such interventions and increase mental well-being, then model 1 would gain empirical support.

In the present paper, we referred mainly to the basic TP dimensions introduced by Zimbardo and Boyd (1999), and only at certain points did we signal extensions to their seminal models, such as future-negative (Carelli et al., 2011). Taking into account the problematic, ambivalent character of the present-hedonistic dimension (see Stolarski et al., 2018), the idea of present-eudaimonic orientation (Vowinckel et al., 2017), characterized by a clearly positive attitude toward the present moment, may confirm particularly illuminating in the search for understanding of the interplay between present focus and savoring.

Moreover, it seems vital to take into account the concept of BTP (e.g., Zimbardo and Boyd, 1999; Zhang et al., 2013) while analyzing the models proposed above. Taking into account its substantial effects on mental well-being, temporal balance may be more important in understanding the role of human temporality than separate TP dimensions. Recent TP conceptualizations have paid much attention to an “optimal” temporal profile that terms the BTP (Zimbardo and Boyd, 1999). The concept emphasizes the key role of effectively switching between particular time horizons in response to situational requirements (Zimbardo and Boyd, 1999; Stolarski et al., 2015b). Such a “temporal flexibility” is said to be crucial for personal effectiveness and adaptation. BTP is thus described as “the mental ability to switch effectively among TPs depending on task features, situational considerations, and personal resources, rather than be biased toward a specific TP that is not adaptive across situations” (Zimbardo and Boyd, 1999, p. 1285). BTP is said to be one of the strongest predictors of mental health (Boniwell et al., 2010; Stolarski et al., 2018). Evidence suggests that BTP correlates positively with subjective well-being and its indicators, such as subjective happiness, positive and negative affect, and vitality (Szcześniak, 2007; Zhang et al., 2013). Interestingly, BTP not only elevates mental well-being but was also shown to buffer negative consequences of maladaptive temperaments on life satisfaction (Stolarski, 2016). BTP, however, was not analyzed in the proposed models; nor were the events in which particular TPs would be more or less beneficial. Further considerations on BTP, WBBs, and well-being can bring more insights into individuals’ choices that would maximize their well-being in a given situation.

What is more, some behavioral features may potentially influence the effects of TPs on well-being. Such behaviors include, for instance, giving and receiving in gratitude intervention conditions (see Watkins, 2014): giving a letter of gratitude to another person was shown to increase well-being significantly (Schueller, 2012). However, giving and receiving a gift abounds with a variety of perceptions, mental states, and conflicting emotions. When done improperly, giving gifts can create insult, hurt, anger, disgrace, remorse, or rage instead of gratitude (Emmons and McCullough, 2003; Wood et al., 2010). This shows that sometimes situational factors may moderate the effects discussed in this paper, causing ambivalent or even negative feelings. To avoid this kind of consequence, it is recommended that both beneficiary and benefactor articulate and commit to an adequate standard (Emmons and McCullough, 2003).

Still little is known about the behavioral mechanisms that can mediate the relationships between TPs and prioritizing positivity. However, high levels of prioritizing positivity were demonstrated to lead to building greater resources such as positive social relations (Catalino et al., 2014). It was also shown that 41.7% of individuals mention interpersonal interactions as contexts, which they use to increase positive emotions when planning pleasant future goals. These results suggest that prioritizing positivity might be related to giving and receiving social support and thus be associated with different temporal perspectives (see Holman and Zimbardo, 2009). The issue definitely seems worth further analysis (see Thomas, 2010).

Any research program aiming to verify the present ideas should also take into account the potential overlap between positive psychology constructs (e.g., Shogren et al., 2006). Although each of the WBBs considered here is shown to be markedly associated with mental well-being, they have scarcely been studied together, and their desirable consequences may in fact be redundant. Secondly, although at the conceptual level the specific temporal anchoring of each WBB seems quite clear, it is possible that, for instance, prioritizing positivity may better explain the positive effects of present-hedonistic on mental well-being than savoring, owing to the particularly high motivation of hedonists to pursue pleasure. Therefore, it seems important to study the associations discussed above within research designs including all TPs in a given study.

Owing to some apparent similarities discussed in the present paper, one may wonder whether TPs and WBBs are really distinct constructs or just different labels for the same phenomena. We believe that the core difference refers to the cognitive versus behavioral nature of these two concepts. Whereas TPs refer mainly to the cognitive processing of ongoing events, including thoughts and perceptions, WBBs depict actual behaviors that are shown to impact happiness and mental well-being robustly. This is also visible in items typically used to measure these constructs (see Table 1 for sample items). The Zimbardo Time Perspective Inventory (ZTPI; Zimbardo and Boyd, 1999) measures five TPs, the scale scores indicating individuals’ tendencies to remain focused on a particular time frame combined with an attitude toward that time horizon. The original version was extended to include a future-negative scale (Carelli et al., 2011). When it comes to the operationalization of WBBs, the Gratitude Questionnaire (GQ6; McCullough et al., 2002) evaluates individual differences in the tendency to experience gratitude in daily life; the Savoring Beliefs Inventory (SBI; Bryant, 2003) provides distinct subscales estimating perceived capacity to savor positive events; and the Prioritizing Positivity questionnaire (Catalino et al., 2014) examines the extent to which individuals search for positive emotional experiences when making decisions about how to organize their day-to-day life. These features of the constructs included in the present models, along with the briefly outlined nature of their psychometric indicators, provide evidence that, despite some theoretical similarities, TPs and WBBs remain both conceptually and empirically distinct phenomena.

**TABLE 1:** Measures of time perspective (TPs) and well-being boosters (WBBs).

	**Measure**	**Sample item**
Time perspectives	Zimbardo Time Perspective Inventory (ZTPI, Zimbardo and Boyd, 1999)	Past-Positive: It gives me pleasure to think about my past. Past-Negative: I think about the bad things that have happened to me in the past. Present-Hedonistic: I take risks to put excitement in my life. Present-Fatalistic: My life path is controlled by forces I cannot influence. Future-Positive: I believe that a person’s day should be planned ahead each morning.
	Future-Negative scale (Carelli et al., 2011)	Future-Negative: To think about my future makes me sad.
**Well-being Boosters**		
Gratitude	Gratitude Questionnaire (GQ6; McCullough et al., 2002)	If I had to list everything that I felt grateful for, it would be a very long list.
Savoring	Savoring Beliefs Inventory (SBI; Bryant, 2003)	It’s easy for me to enjoy myself when I want to.
Prioritizing Positivity	Prioritizing Positivity questionnaire (Catalino et al., 2014)	I structure my day to maximize my happiness.

## Conclusion

The leading focus of this paper was to analyze possible relationships among TPs and gratitude, savoring, and prioritizing positivity, and their interplay in shaping mental well-being. Our central idea is that each of these WBBs has a marked temporal character and thus may be more common and/or effective among individuals manifesting elevated levels of the respective TP dimension. We proposed four hypothetical models that illustrate potential patterns of dynamics among TPs, WBBs, and mental well-being. The first model, referred to as the trait-behavior model, proposes that trait TPs impact respective WBBs and that WBBs mediate between TPs and mental well-being. The second model, labeled the accumulation model, assumes opposite causality, claiming that frequently using particular WBBs may lead to gradual changes in respective TPs, consequently leading to elevation in mental well-being. The third model, termed the feedback loop, suggests that actually, both of these seemingly opposite processes act in parallel: WBBs and TPs strengthen one another, and that process fosters mental well-being. Finally, the match–mismatch model proposes that the effect of WBBs on mental well-being may be moderated by TPs, suggesting that a correspondence between temporal anchoring of a given WBB and high levels of the respective TP may enhance the impact of the former on mental well-being.

We believe that the present article provides a useful basis for complex experimental analyses of the temporal nature of different strategies aiming to increase mental well-being. It seems plausible that data gathered in future research aiming to test the present hypothetical models will allow researchers to maximize their effectiveness, for example, via personalizing the set of positive interventions in a way that corresponds to individuals’ TP profiles. Positive intervention programs refer to a systematic approach, where the aim is defeating one’s challenges by using psychological resources and character strengths during a regular practice that brings sustainable effects (Seligman et al., 2006). In light of the present consideration, TPs, particularly the positive ones, may be treated as a type of vital resource that allows an individual to develop greater levels of mental well-being.

## Author Contributions

BB and MS developed the conceptual models. BB drafted the initial version of the manuscript. MS drafted the parts about the concept of time perspectives and revised the initial version of the manuscript.

## Conflict of Interest

The authors declare that the research was conducted in the absence of any commercial or financial relationships that could be construed as a potential conflict of interest.
